# Supplementary material for: Thought experiment: Decoding cognitive processes from the fMRI data of one individual
Source: PLoS One. 2018 Sep 20;13(9):e0204338. doi: 10.1371/journal.pone.0204338 (PMC6147600; doi:10.1371/journal.pone.0204338)
Supplement: S1 File — Reference guide for instructing a participant to perform the five mental imagery tasks. (PDF) [file pone.0204338.s001.pdf]

Reference guide for instructing participants

Language

If we tell you a certain keyword, e.g. 'fruits', try to come up with as many names of different fruits as you can think of. Can you tell me what possible kinds of fruits you could come up with?

Sensory-motor skills

Imagine playing a sport, e.g. tennis. Imagine how you would move if you were playing that sport. Concentrate on what your limbs would do. Try to keep occupied throughout.

Visuo-spatial memory

Imagine walking to a certain location, for example the train station. You can pick your home as a starting location or, if the destination is too far away from your home, a place nearby. Concentrate on the street, the buildings and landmarks you would use to navigate to the respective destination. Pace yourself so you will not get there too quickly. In case you reach the destination before 30 seconds have passed, don't stop there but keep moving around.

Visual processing of faces

Imagine the faces of persons you know. Concentrate on the inner part of their faces, i.e. eyes, nose and mouth. If a certain face gets boring or is hard to visualize, switch to another face. For example, if we ask you to imagine movie actors, try to imagine the faces of a number of different actors during the course of the 30 seconds. Switch to the face of a different actor every once in a while.

Resting

Try to stop the task you were engaged in before, even if you find this hard to do. Try to think of nothing in particular and if a certain thought enters your mind, let it go. Don't worry if you're not always able to do so. Try to stay relaxed but stay awake throughout.
