# Supplementary material for: Thought experiment: Decoding cognitive processes from the fMRI data of one individual
Source: PLoS One. 2018 Sep 20;13(9):e0204338. doi: 10.1371/journal.pone.0204338 (PMC6147600; doi:10.1371/journal.pone.0204338)
Supplement: S2 File — Full study design with onsets and durations of all blocks. (PDF) [file pone.0204338.s002.pdf]

run001

time	volume	domain	content
00:00	0	rest	rest
00:30	10	language	animals
01:00	20	spatial	city square
01:30	30	faces	family
02:00	40	motor	badminton
02:30	50	language	tools
03:00	60	faces	friends
03:30	70	spatial	market
04:00	80	rest	rest
04:30	90	motor	tennis
05:00	100	spatial	tram station
05:30	110	language	cities
06:00	120	faces	movie actors
06:30	130	rest	rest
07:00	140	motor	swimming
07:30	150	faces	athletes
08:00	160	spatial	train station
08:30	170	language	countries
09:00	180	rest	rest
09:30	190	motor	soccer
10:00	200	language	occupations
10:30	210	spatial	school
11:00	220	rest	rest
11:30	230	faces	TV actors
12:00	240	motor	high jump

run002

time	volume	domain	content
00:00	0	spatial	church
00:30	10	rest	rest
01:00	20	language	fruits
01:30	30	faces	politicians
02:00	40	motor	climbing
02:30	50	rest	rest
03:00	60	spatial	basement
03:30	70	faces	lecturers
04:00	80	language	clothing
04:30	90	motor	hurdle race
05:00	100	faces	teachers
05:30	110	rest	rest
06:00	120	spatial	promenade
06:30	130	language	vegetables
07:00	140	motor	archery
07:30	150	spatial	city of Kiel
08:00	160	faces	family
08:30	170	rest	rest
09:00	180	language	furniture
09:30	190	motor	rope skipping
10:00	200	rest	rest
10:30	210	faces	friends
11:00	220	language	colors
11:30	230	spatial	university
12:00	240	motor	juggling

run003

time	volume	domain	content
00:00	0	faces	movie actors
00:30	10	language	countries
01:00	20	rest	rest
01:30	30	spatial	market
02:00	40	motor	badminton
02:30	50	language	animals
03:00	60	rest	rest
03:30	70	faces	athletes
04:00	80	spatial	school
04:30	90	motor	swimming
05:00	100	rest	rest
05:30	110	language	animals
06:00	120	spatial	city square
06:30	130	faces	family
07:00	140	motor	badminton
07:30	150	language	tools
08:00	160	faces	friends
08:30	170	spatial	market
09:00	180	rest	rest
09:30	190	motor	tennis
10:00	200	spatial	church
10:30	210	rest	rest
11:00	220	language	fruit
11:30	230	faces	politicians
12:00	240	motor	climbing
